# A New Removable Uterine Compression by a Brace Suture in the Management of Severe Postpartum Hemorrhage

**DOI:** 10.3389/fsurg.2014.00043

**Published:** 2014-11-17

**Authors:** Abderrahim Aboulfalah, Bouchra Fakhir, Yassir Ait Ben Kaddour, Hamid Asmouki, Abderraouf Soummani

**Affiliations:** ^1^Department of Gynecology and Obstetrics, University Hospital Mohammed VI, Marrakech, Morocco

**Keywords:** postpartum hemorrhage, surgical management, uterine compressive sutures, uterine brace compressive sutures, conservative treatment

## Abstract

Postpartum hemorrhage (PPH) is a life-threatening complication of delivery. It is the leading cause of maternal mortality. During the last 15 years, several total uterine compressive sutures were described in literature. They have proven their effectiveness and safety in the management of severe PPH as an alternative to hysterectomy. We present in this paper a new technique of uterine compressive sutures based on removable uterine brace compressive sutures with compression of the uterus against the pubis. This technique may be more effective by using two mechanisms of uterine bleeding control and also may prevent uterine synechia by respecting the uterine cavity and the removal of the suture 1 or 2 days later. We also present the results of a 15 patients’ series using this new suture.

## Introduction

Postpartum hemorrhage (PPH) is a life-threatening complication of delivery. It is the leading cause of maternal death (1), with 1 to 13% of births around the world affected (2). In Morocco PPH was around 132/100,000 in 2009 were documented with PPH complication. It occurs in approximately 4% of vaginal deliveries and 6% of cesarean deliveries (3). Definition of PPH differs between authors; in general, it is a loss of more than 500 ml of blood after vaginal delivery or 1000 ml after cesarean section (2). Severity criteria are uncontrolled bleeding after initial medical management of PPH, hemodynamic instability even resuscitation by crystalloids and red blood cells, and presence of coagulation disturbances. Surgery is then indicated. Since the first publication of B-lynch technique in 1997 (4), different uterine compression sutures have been described and performed as an alternative to hysterectomy. Based on compressive sutures, they have proven to be valuable and safe in the control of massive PPH (5). Recently, uterine synechia has been reported as a frequent complication of those sutures, with 18–54% frequency, which surely compromises fertility (6, 7). Sutures that run through the full thickness of both anterior and posterior uterine walls and infection are involved in this complication.

In order to prevent synechia and using two uterine bleeding control mechanisms, we conceptualized and performed from 2006 to 2011 a new uterine compressive brace suture procedure (initially described by the first author), in 15 patients with severe PPH, in the gynecology obstetrics department of Mohammed VI university hospital, Marrakech, Morocco.

## Materials and Methods

### Surgical technique description

The principle of the technique is a removable uterine brace suture, which compresses uterus against the pubis.

**Figure 1:**
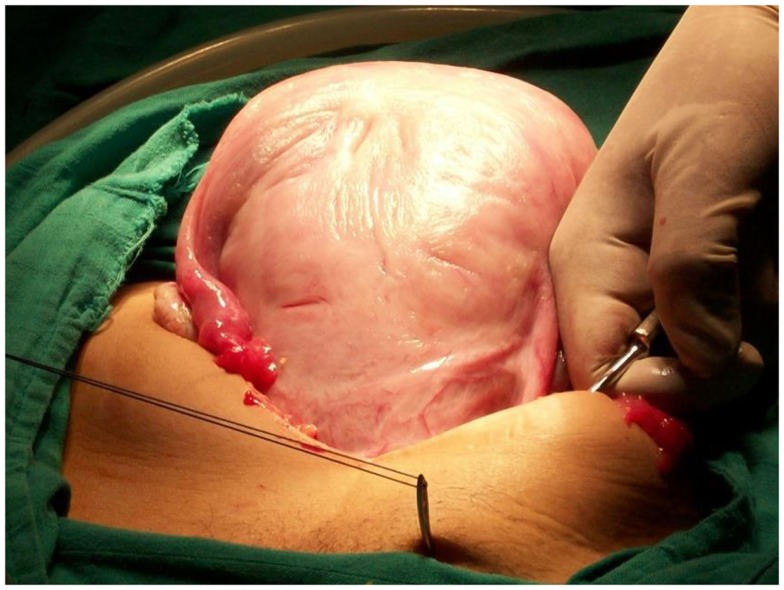
**First cutaneous stitch**.

**Figure 2:**
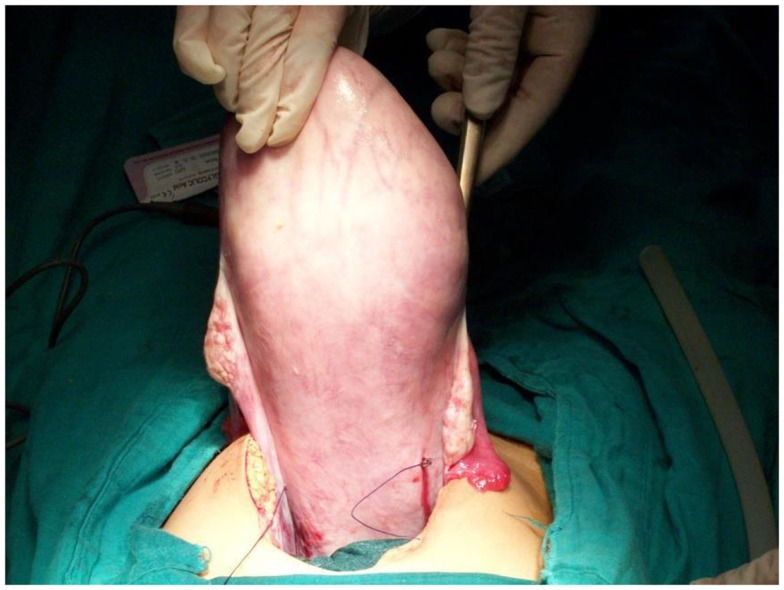
**Second uterine stitch, posterior view**.

**Figure 3:**
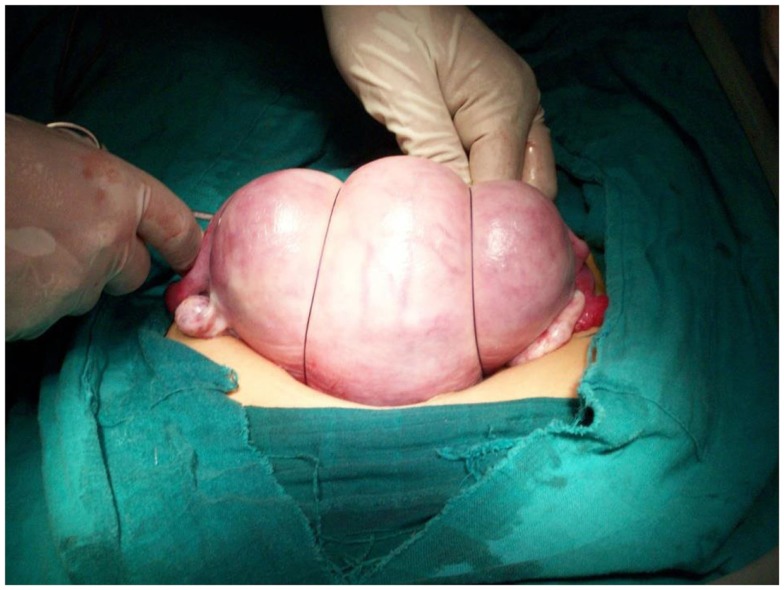
**Brace position of the sutures**.

**Figure 4:**
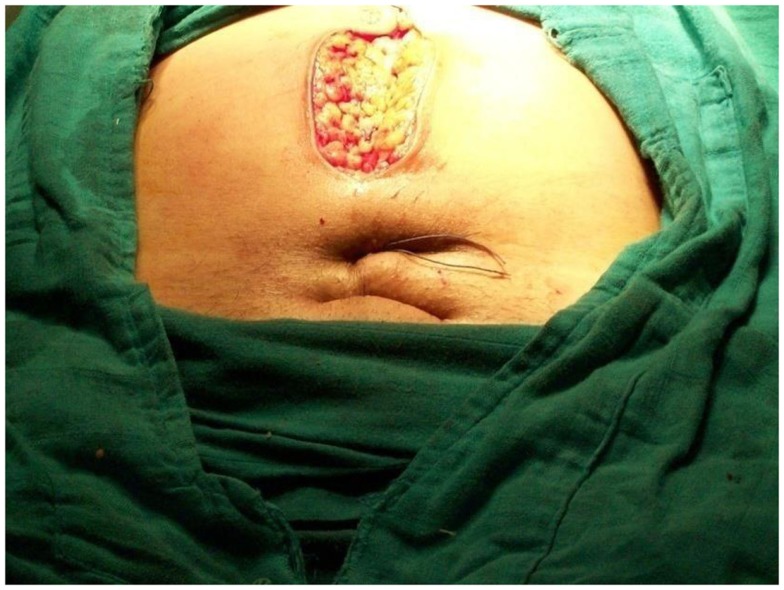
**Final cutaneous position of suture**.

Under general anesthesia, the patient is placed in the Lloyd Davis position, a urinary catheter in place, and an assistant is positioned between the patient’s legs to assess vaginal bleeding. The same incision as for a cesarean section can be used or if PPH occurs after vaginal delivery, both below umbilical median or transversal incisions can be performed. After uterine exteriorization and pelvic exploration, a test is carried out to assess the effectiveness. We proceed by front curving and compressing the uterus against the pubis, if bleeding has decreased or stopped, the procedure has a high chance of stopping PPH. First, the bladder peritoneum is reflected inferiorly. Using a number 2 sliding non-resorbable suture wire with 70 mm round-bodied hand needle or with wire guide, the first stitch is applied from outside, running through the full thickness of anterior abdominal wall above the pubis immediately and 2 cm laterally from the median line. Starting from the right side or left side is the same. After that, the needle is passed through the inferior uterine segment from the anterior to posterior wall as low as possible, under sutured hysterotomy, and 2 cm inside from uterine artery cross. The wire is then passed over as a brace to compress the uterine fundus by approximately 3 or 4 cm inside the corneal border. Finally, the last stitch is applied from inside to outside through the abdominal wall 2 cm above the first parietal stitch but 4 cm laterally from the median line. The same procedure is realized from the other side of the median line. Finally, the right and the left lower suture extremities are tied anteriorly, followed by the upper extremities with added curves and compression of the uterus against the pubis (Figures 1–4). The throws are visible to the skin. Efficacy is immediately checked. Twenty-four to forty-eight hours later maximum, the throws are cut, and sutures are removed by simple wire traction without any anesthesia.

### Population characters

Initially, the technique was used with 12 primiparous patients with severe PPH, defined as uncontrolled bleeding after initial uterotonic medical management, with hemodynamic instability even after resuscitation by crystalloids and red blood cells and after vascular ligature. The purpose was to prevent hysterectomy. Following the initial success, the technique was performed in 3 cases immediately after medical management failure.

## Results

In our 15 procedures, as described in Table 1, PPH occurred in 11 cases (73%) after vaginal delivery and in 4 cases (27%) after cesarean section. Eighty percent were caused by uterine atony. In 11 cases, the technique was realized secondarily after vascular ligature failure alone, and in 1 case after partial uterine resection for accret placental. One hundred percent of hemostasis was obtained; one (7%) secondary hysterectomy was done for bleeding relapse 3 h later. One death occurred secondary to preeclampsia with cerebral vascular accident. No particular complications were noted. During post-operative follow up, all patients regained their normal menstrual cycles. Five pregnancies were attempted, and three normal pregnancies were achieved.

**Table 1:** **Population characters and technique results**.

	*n* (**%)**
Mean age	25 years
Primiparous	12 (80%)
Second parous	3 (20%)
Vaginal delivery	11 (73%)
Cesarean section	4 (27%)
Severe PPH	15 (100%)
Blood resuscitation	15 (100%)
Hemodynamic instability	15 (100%)
**PPH etiology**
Uterine atony	12 (80%)
Accret placental	2 (13%)
Uterine rupture	1 (7%)
Secondary hysterectomy	1(7%)
Maternal mortality	1 (7%)
Mean time duration of procedure	14 min
Blood loss	2300 ml +/− 500 ml

## Discussion

We describe an innovative method, which is simple, effective, easy to learn, tried with successful outcome for the control of severe PPH as an alternative to more complicated surgeries like hysterectomy. This technique uses two mechanisms of bleeding control by compression of placental site by tight compression of uterine walls and by obstruction of blood flow through uterine arteries by extreme forward flection of the uterus.

Preventing uterine synechia is possible because uterine cavity is respected, there is no need to open the cavity by a new hysterotomy compared to original B-Lynch suture (4–8), and suture does not pass through the full thickness of both anterior and posterior uterus body wall compared to cho sutures (9), or to compressing *U*-sutures (10). Also it is known that inflammation around sutures and infection are responsible of synechia. So, the most innovative particularity of our technique is the removal of the suture 24 or 48 h later. This is the first time that a compressing uterine suture technique is followed by removal of the suture, and these three details may be the key of preventing synechia by decreasing the risk of infection. There is no foreign body inside the uterine cavity, and spontaneous cervical drainage is done after suture removal; hence, pyometra is avoided. Also, removing sutures prevents joining of endometrial walls over time, which itself increases the risk of infection.

## Conclusion

Removable uterine brace compressive against pubis suture is a promising technique, simple, safe, and effective in management of severe PPH, adapted to most of PPH causes, from uterine atony to placenta accreta. It may prevent synechia and help maintain fertility by respecting uterine cavity and this more precisely by percutaneous removing of the suture 24–48 h later. We think that this technique deserves to be applied in greater number with added systematic hysteroscopy control to prove its superiority in synechia prevention.

## Conflict of Interest Statement

The authors declare that the research was conducted in the absence of any commercial or financial relationships that could be construed as a potential conflict of interest.
